# The Population-Based Risk of Need for Coronary Revascularization According to the Presence of Type 2 Diabetes Mellitus and History of Coronary Heart Disease in the Korean Population

**DOI:** 10.1371/journal.pone.0128627

**Published:** 2015-06-08

**Authors:** Chang Hee Jung, Gi Hyeon Seo, Sunghwan Suh, Ji Cheol Bae, Mee Kyoung Kim, You-Cheol Hwang, Jae Hyeon Kim, Byung-Wan Lee

**Affiliations:** 1 Department of Internal Medicine, Asan Medical Center, University of Ulsan College of Medicine, Seoul, Korea; 2 Health Insurance Review and Assessment Service, Seoul, Korea; 3 Department of Internal Medicine, Dong-A University Medical Center, Busan, Korea; 4 Department of Medicine, Samsung Medical Center, Sungkyunkwan University School of Medicine, Seoul, Korea; 5 Department of Internal Medicine, The Catholic University of Korea, Seoul, Korea; 6 Department of Internal Medicine, Kyung Hee University School of Medicine, Seoul, Korea; 7 Department of Internal Medicine, Severance Hospital, University of Yonsei University College of Medicine, Seoul, Korea; Nagoya University, JAPAN

## Abstract

**Background:**

Whether diabetic patients without a history of coronary heart disease (CHD) have the same risk of CHD events as non-diabetic patients with a history of CHD remains controversial. This study aimed to determine whether type 2 diabetes mellitus (T2DM) is a coronary heart disease (CHD) equivalent in the need for coronary revascularization procedures (RVs) in the Korean population.

**Methodology/Principal Findings:**

We followed 2,168,698 subjects who had oral anti-diabetic drugs (OADs)-taking T2DM in 2008 and/or CHD in 2007–2008 (i.e., recent CHD). We used systematic datasets from the nationwide claims database of the Health Insurance Review and Assessment service of Korea, which is representative of the whole population of Korea, from January 2007 to December 2012. The primary study endpoint was the development of need for RVs (i.e., incident CHD) after January 2009 among three groups based on their status of T2DM and recent CHD, i.e., T2DM only, recent CHD only, and both T2DM and recent CHD. After adjustment for age and sex, patients with recent CHD only had 2.14 times the risk of incident CHD (95% CI, 2.11–2.18, *P*<0.001) compared with patients with T2DM only. Patients with both T2DM and recent CHD demonstrated approximately 2-fold increased risk of incident CHD compared with subjects with recent CHD only (95% CI, 1.75-1.82), while 4-fold increased risk compared with subjects with T2DM only (95% CI, 3.71-3.87). The risk of incident CHD also differed according to sex and age.

**Conclusions/Significance:**

This analysis of data from the nationwide claims database revealed that T2DM did not have a recent CHD equivalent risk in the Korean population. These results suggest that an appropriate strategy for the CHD risk stratification in diabetic patients should be adopted to manage this population.

## Introduction

Although type 2 diabetes mellitus (T2DM) is a well-known risk factor for cardiovascular disease (CVD) and is associated with a 2- to 4-fold increase in the risk of developing coronary heart disease (CHD) [1–3], the magnitude of that risk recently faces challenges. In 1998, Haffner SM et al. demonstrated that subjects with T2DM but without prior myocardial infarction (MI) had a risk for fatal cardiovascular events equivalent to subjects without T2DM who had survived an MI [4].

Since the landmark study by Haffner SM et al. described above, numerous reports have also supported the concept of diabetes as a CHD risk equivalent [5–10], while others have refuted it [11–16].As a result of these inconsistent outcomes, a meta-analysis was recently performed to further test the concept of diabetes as a CHD equivalent [17]. The analysis did not support the hypothesis that diabetes is a CHD equivalent and suggested that new appropriate patient CHD risk estimates should be adopted in treating patients with T2DM rather than a ‘blanket’ approach of cardio protective drugs [17]. However, most studies were based mainly on Caucasian populations, and there has been minimal research in Asian populations, including in Korea. There are striking ethnic differences in CHD risk [18]. Furthermore, Asian countries including Korea are emerging as the epicenter of the epidemic of diabetes due to their large populations and rapid economic growth [19]. As the prevention and treatment of CHD is an enormous medical and socio-economic problem, this issue of T2DM as a CHD equivalent is of considerable importance from an economic perspective as well as from a therapeutic perspective.

The present study aimed to clarify whether T2DM is a recent CHD equivalent in the need for coronary revascularization procedures (RVs) in a Korean population through analysis of nationwide population data from the Korean National Claim Registry in which all Koreans are obliged to enroll.

## Subjects and Methods

### Data sources

Systematic datasets from the nationwide claims database of the Health Insurance Review and Assessment (HIRA) service of Korea from January 2007 to December 2012 were used for the analysis. In Korea, 97% of the population is obliged to enroll in the Korean National Health Insurance Program. Patients pay approximately from 5% to 30% of total medical costs to clinics or hospitals, although some services are not covered by insurance, such as cosmetic surgery and some unproven therapies. Clinics and hospitals then submit claims for inpatient and outpatient care, including data on diagnoses [as determined by the International Classification of Diseases, 10^th^ revision (ICD-10)], procedures, prescription records, demographic information, and direct medical costs, to obtain reimbursement for total medical costs (ranged from 70% to 95%). The remaining 3% of the population not insured by the Korean National Health Insurance Program are either covered by another Medical Aid Program or are temporary or illegal residents. These claims are also reviewed by HIRA, and thus, virtually all information pertaining to patients and their medical records is available in the Korean HIRA database. The cohort of patients aged 40 to 79 years in January 2009 was recruited from the HIRA service database, which provided a representative dataset of the whole population of Korea. This dataset has been previously used to conduct epidemiological studies in Korea [20–22], and the background and configuration process of this dataset was described elsewhere in detail [23]. As all data were analyzed anonymously, consent was not specifically obtained. The institutional review board of the Asan Medical Center (Seoul, Republic of Korea) approved this study (IRB No. 2014–0815).

### Definition of prevalent T2DM

T2DM was defined as being prevalent if subjects were taking oral anti-diabetic drugs (OADs) for more than 90 days in the year of 2008. T2DM was further classified into two categories: ‘new-onset T2DM’ if there were no claims in the database relating to OADs in 2007 and ‘established T2DM’ in the remaining cases.

### Ascertainment of recent CHD and need for coronary RVs

The primary study endpoint was the development of need for coronary RVs after January 2009 among three groups based on their status of T2DM and recent CHD. The three groups were T2DM only, recent CHD only, and both T2DM and recent CHD. We ascertained the status of recent CHD using records of the Korean HIRA database from 2007 to 2008; earlier health records could not be obtained due to the abrogation of the database according to government policy. We defined recent CHD based on the codes for coronary RVs including percutaneous coronary intervention (PCI) and/or coronary artery bypass graft (CABG). One or more procedure codes of PCI (M6551-2, M6561-4, and M6571-2) and CABG (O1641-2, O1647, OA641-2, and OA647) were required for inclusion in this study. In addition, recent CHD was defined by using the hospital discharge databases of the HIRA service [ICD-10 codes I20 (angina pectoris), I21 (ST elevation and non-ST elevation myocardial infarction), I22 (subsequent ST elevation and non-ST elevation myocardial infarction), I23 (certain current complications following ST elevation and non-ST elevation myocardial infarction), I24 (other acute ischemic heart diseases), and I25 (chronic ischemic heart disease)] and/or more than two outpatient visits per year due to CHD defined by using data from the HIRA (ICD-10 codes ranged from I20 to I25) as a principal diagnosis.

To identify the need for RVs, we followed each patient by above procedure codes of PCI and/or CABG from January 2009 until December 2012. In subjects suffering from multiple coronary events, the first event was considered to be the need for RVs.

### Statistical analysis

Continuous and categorical variables are represented as the mean ± standard deviation (SD) and as percentage (%), respectively. Baseline characteristics among the three group (T2DM only, recent CHD only, and both T2DM and recent CHD) were compared using one-way analysis of variance (ANOVA) with Tukey’s method as the post-hoc analysis for continuous variables and the chi-squared test for categorical variables.

To estimate incidence rates of need for RVs according to the presence of T2DM and/or recent CHD, we calculated actual event rates per 100,000 person-years. We calculated and compared the cumulative incidences of need for RVs by the Kaplan–Meier method, and statistical differences among three groups according to the presence of T2DM and/or recent CHD were compared by the log-rank test. Statistical differences among groups were compared by calculating the hazard ratios (HRs) and 95% confidence intervals (CIs) for the need for RVs according to the presence of T2DM and/or recent CHD. Multivariate Cox proportional hazard models were applied after adjustment for age and gender. Statistical analyses were conducted using R version 2.15.3 (R Foundation for Statistical Computing, Vienna, Austria, http://www.R-project.org). All tests were two-sided, and *P*<0.05 was considered significant.

## Results

A total of 2,168,698 subjects (mean age 61.4 ± 9.9 years, male 52.8%) who had T2DM in 2008 and/or CHD in 2007–2008 (i.e., recent CHD) were enrolled and followed up for the development of need for RVs. The total follow-up period was 8,511,148 person-years and the average follow-up period was 3.92 years. During the study period, 74,343 (3.4%) subjects developed the need for RVs. The event rate (i.e., need for RVs) in this population was 873.5 per 100,000 person-years. The cohort was classified into three groups based on their status of T2DM and recent CHD at baseline: T2DM without recent CHD (group I, henceforth “T2DM only”), recent CHD without T2DM (group II, henceforth “recent CHD only”), and recent CHD with T2DM (group III, henceforth “both T2DM and recent CHD”). The mean age and gender distribution at baseline are shown in Table 1. The subjects in group III were significantly older and there was a higher proportion of men (Table 1).

**Table 1:** Baseline characteristics of the study subjects by presence or absence of T2DM and recent CHD.

	Group I	Group II	Group III	*P* value
**Number**	1,544,892	495,694	128,112	-
**Age (years)**	60.9±9.9	62.1±10.0	64.5± 8.8	<0.001^a^
**Men (%)**	52.7	52.7	54.9	<0.001^b^

Group I, II and III represent subjects with T2DM only, recent CHD only, and both T2DM and recent CHD, respectively.

^a^Analyzed using ANOVA.

^b^Analyzed using chi-squared test.

**Fig 1:**
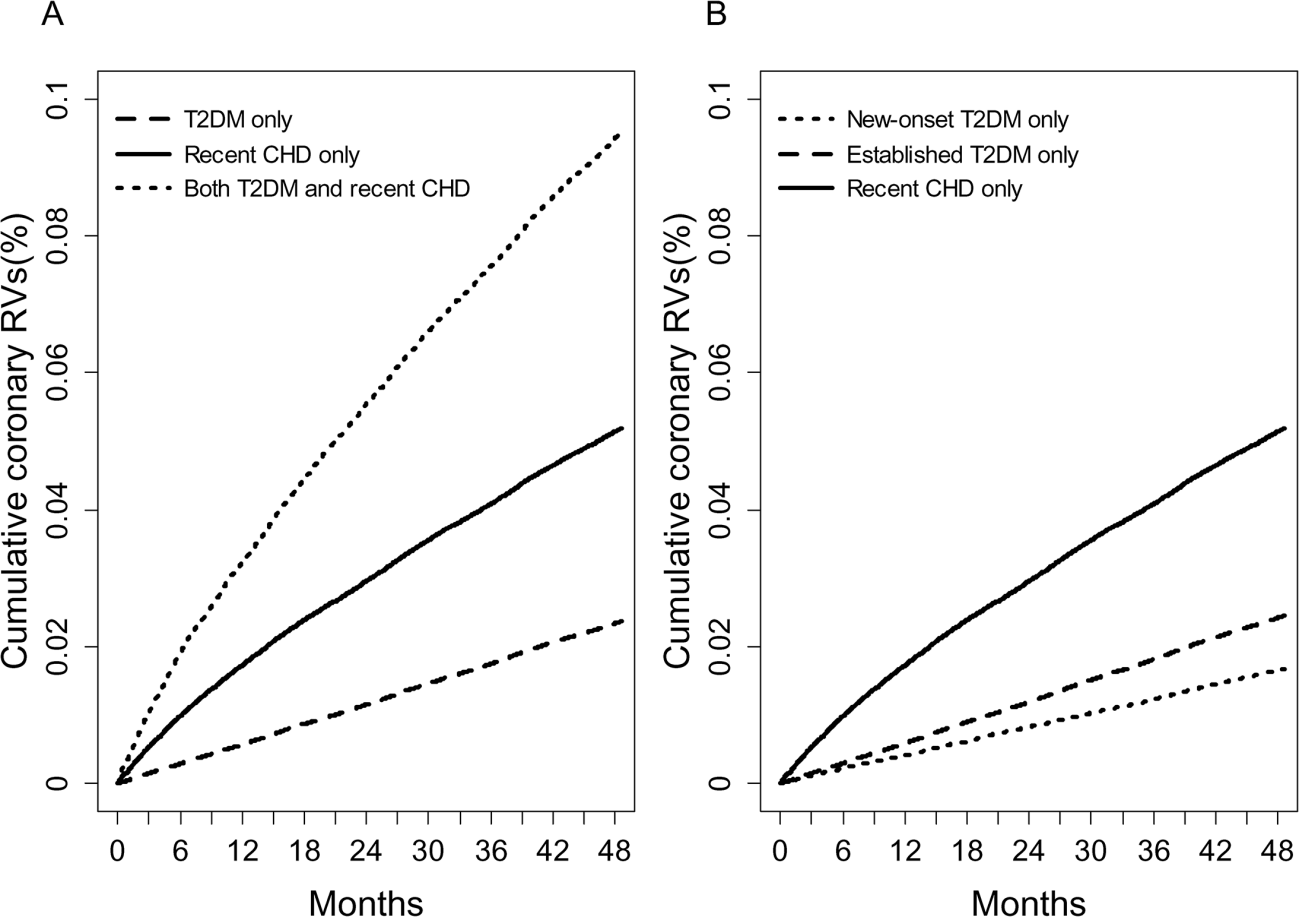
(a) The cumulative incidence of the need for RVs by presence or absence of T2DM and recent CHD (log-rank test, *P*<0.001 for all comparisons) and (b) according to the duration of T2DM (new-onset T2DM vs. established T2DM) and recent CHD (log-rank test, *P*<0.001 for all comparisons).

Fig 1A shows the cumulative incidences of need for RVs according to the three groups. The incidence rates of need for RVs in group I, II and III were 597.9, 1334.6, and 2513.5 per 100,000 person-years, respectively.

Table 2 shows the HRs and 95% CIs of the need for RVs by presence or absence of T2DM and recent CHD at baseline. Compared to group I, group II and group III showed significantly higher HRs for need for RVs. Although the significance was slightly lower after adjustment for age and sex, the adjusted HRs (95% CI) were still significantly higher in group II and group III than in group I (Table 2).

**Table 2:** Hazard ratios (HRs) for the need for RVs by presence or absence of T2DM and recent CHD.

	Group I	Group II	Group III
**Incidence rate** ^**a**^	597.9	1334.6	2513.5
**Crude HR**	Ref.	2.23 (2.20–2.27)	4.20 (4.11–4.29)
**Age- and sex-adjusted HR**	Ref.	2.14 (2.11–2.18)	3.79 (3.71–3.87)
**Crude HR**	-	Ref.	1.88 (1.84–1.92)
**Age- and sex-adjusted HR**	-	Ref.	1.79 (1.75–1.82)

^a^per 100,000 person-years.

Group I, II and III represent subjects with T2DM only, recent CHD only, and both T2DM and recent CHD, respectively.

*P*<0.001 for all HRs.

Fig 1B shows the cumulative incidences of need for RVs as a function of the duration of T2DM (new-onset T2DM vs. established T2DM) and the presence of recent CHD. The incidence rate of need for RVs in group I with new-onset T2DM, group I with established T2DM and group II were 422.0, 619.2, and 1334.6 per 100,000 person-years, respectively (Table 3). The age- and sex-adjusted HR (95% CI) for incident CHD in group I with established T2DM was 1.36 (1.31–1.42) compared with group I with new-onset T2DM. However, group II subjects still showed significantly higher HRs compared to either type of group I [age- and sex-adjusted HRs (95% CI); 2.95 (2.83–3.06) compared with group I with new-onset T2DM and 2.08 (2.05–2.12) compared with group I with established T2DM, Table 3].

**Table 3:** Hazard ratios (HRs) for the need for RVs according to the duration of T2DM and recent CHD.

	Group I with new onset T2DM	Group I with established T2DM	Group II
**Incidence rate** ^**a**^	422.0	619.2	1334.6
**Crude HR**	Ref.	1.47 (1.41–1.53)	3.16 (3.04–3.28)
**Age- and sex-adjusted HR**	Ref.	1.36 (1.31–1.42)	2.95 (2.83–3.06)
**Crude HR**	-	Ref.	2.15 (2.12–2.19)
**Age- and sex-adjusted HR**	-	Ref.	2.08 (2.05–2.12)

^a^per 100,000 person-years.

Group I, and II represent subjects with T2DM only, and recent CHD only, respectively.

*P*<0.001 for all HRs.

Table 4 shows the HRs and 95% CIs of need for RVs by presence or absence of T2DM and recent CHD, defined by the various combinations of hospital discharge databases of the HIRA (i.e., ICD-10 codes ranged from I20 to I25): more than two outpatient visits per year due to CHD, defined using data from the HIRA (ICD-10 codes ranged from I20 to I25) as a principal diagnosis; and/or codes related with coronary RVs (PCI and/or CABG) at baseline. Regardless of the definition used for recent CHD, the risk of need for RVs was in the following order, from lowest to highest: group I, group II and group III.

**Table 4:** Hazard ratios (HRs) for the need for RVs by presence or absence of T2DM and recent CHD defined using various definitions.

	Group I	Group II	Group III
	**Recent CHD defined by the ICD-10 codes (I20- I25)**		
**Incidence rate** ^**a**^	597.9	1088.9	2144.3
**Crude HR**	Ref.	1.82 (1.79–1.85)	3.59 (3.50–3.67)
**Age- and sex-adjusted HR**	Ref.	1.76 (1.73–1.80)	3.22 (3.15–3.30)
**Crude HR**	-	Ref.	1.97 (1.92–2.02)
**Age- and sex-adjusted HR**	-	Ref.	1.83 (1.78–1.88)
	**Recent CHD defined by the ICD-10 codes (I20 only)**		
**Incidence rate** ^**a**^	597.9	980.8	2051.3
**Crude HR**	Ref.	1.64 (1.61–1.67)	3.43 (3.33–3.53)
**Age- and sex-adjusted HR**	Ref.	1.63 (1.59–1.66)	3.11 (3.03–3.20)
**Crude HR**	-	Ref.	2.09 (2.02–2.16)
**Age- and sex-adjusted HR**	-	Ref.	1.90 (1.84–1.96)
	**Recent CHD defined by the ICD-10 codes (I21- I25)**		
**Incidence rate** ^**a**^	597.9	1371.2	2327.8
**Crude HR**	Ref.	2.29 (2.23–2.35)	3.89 (3.75–4.04)
**Age- and sex-adjusted HR**	Ref.	2.10 (2.04–2.15)	3.38 (3.25–3.51)
**Crude HR**	-	Ref.	1.70 (1.62–1.77)
**Age- and sex-adjusted HR**	-	Ref.	1.64 (1.57–1.72)
	**Recent CHD defined by the coronary RVs**		
**Incidence rate** ^**a**^	597.9	3357.1	4109.9
**Crude HR**	Ref.	5.61 (5.47–5.75)	6.87 (6.64–7.10)
**Age- and sex-adjusted HR**	Ref.	4.94 (4.82–5.07)	6.01 (5.81–6.22)
**Crude HR**	-	Ref.	1.22 (1.17–1.27)
**Age- and sex-adjusted HR**	-	Ref.	1.24 (1.19–1.29)

^a^per 100,000 person-years.

Group I, II and III represent subjects with T2DM only, recent CHD only, and both T2DM and recent CHD, respectively.

*P*<0.001 for all HRs.

**Fig 2:**
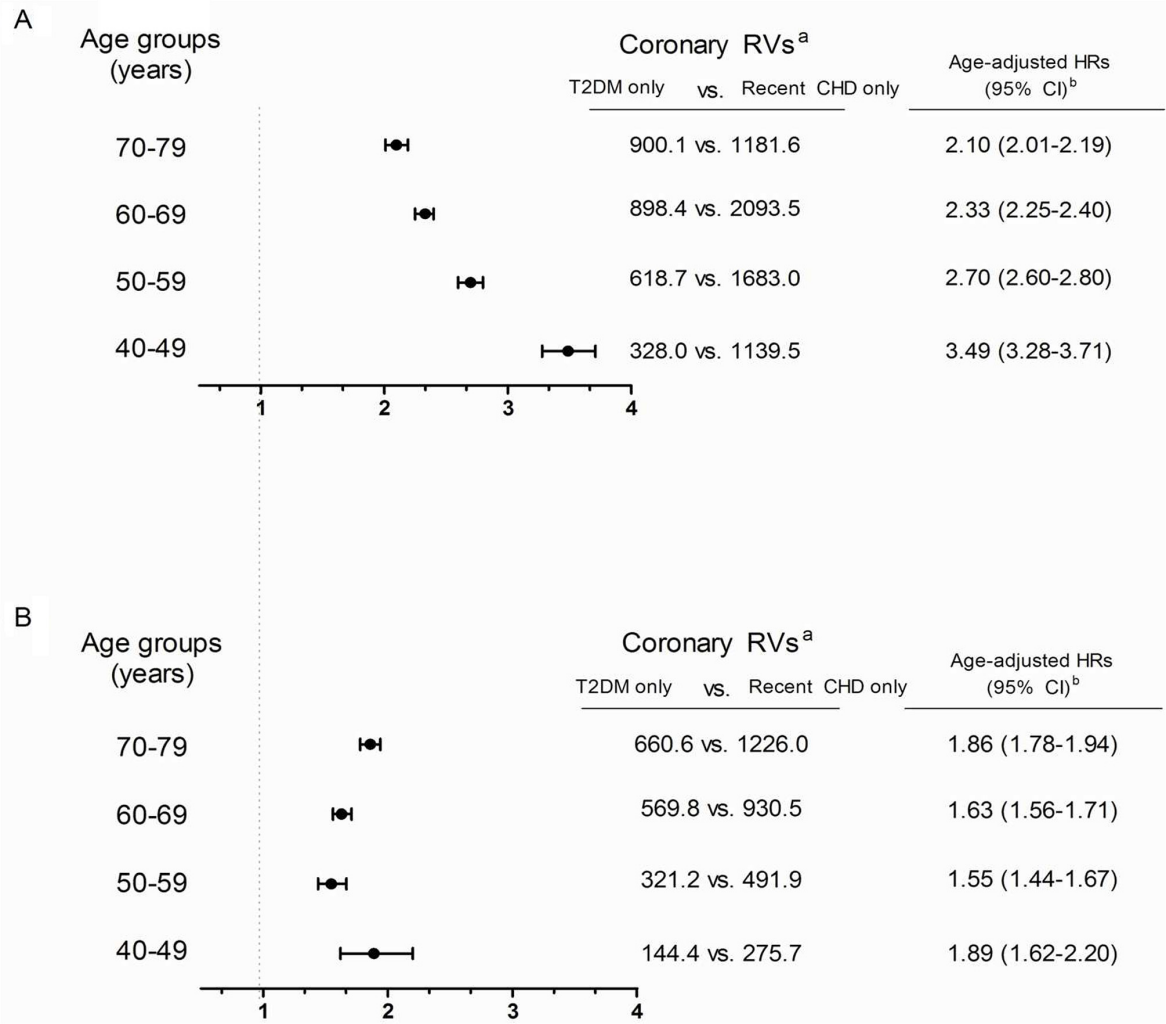
Age-adjusted hazard ratios (HRs) for the need for RVs in subjects with T2DM only vs. recent CHD only in (a) men and (b) women (*P*<0.001 for all HRs). ^a^per 100,000 person-years. ^b^Ref. group is T2DM only group.

Fig 2 shows the rate of need for RVs and age-adjusted HRs (95% CI) for need for RVs according to the various age groups and sex. Overall, the age-adjusted HRs in the recent CHD only group for need for RVs (compared with T2DM only group) were lower in women than in men across all age groups (Fig 2). Regarding the risk according to age, the risk in the T2DM only group approached that of the recent CHD only group at higher ages in men. For women, the risk gap between two groups (T2DM only vs. recent CHD only) was smallest in 50–59 year age group (Fig 2). However, the need for RVs in the T2DM only group increased as age increased in both sexes (Fig 2).

## Discussion

In this nationwide population-based cohort study, we investigated whether diabetic persons without a recent history of CHD had a recent CHD equivalent risk for need for RVs as non-diabetic persons with a recent history of CHD. We found that a prior history of CHD conferred a higher risk than diabetes alone, and the results depended on sex and age. Our data also demonstrated a 2-fold increased risk of need for RVs in subjects with both T2DM and recent CHD compared with subjects with recent CHD only, while their 4-fold increased risk compared with subjects with T2DM only (Table 2). This finding highlights the importance of aggressive secondary prevention in patients with coexisting T2DM and CHD.

There are few controversies about the increased incidence of CHD and cardiovascular morbidity and mortality in subjects with T2DM [1–3]. However, it is unclear whether subjects with T2DM who have not had CHD have an equivalent risk of future CHD compared to those without T2DM who have had a CHD. This is an important issue in light of the aggressive multi-factorial strategies that are conventionally implemented to decrease patient risk factors for CHD [17]. Furthermore, this concept might not be translated into the Korean population, since the prevalence and incidence of CHD are significantly lower than in Caucasians [24,25].

Recently, Kim et al. found no relationship between death from CVD and diabetes in three well-established population-based cohort studies (n = 3,801) in Korea [26]. Based on these facts, we hypothesized that the need for RVs in Korean subjects with T2DM without CHD might not be a recent CHD equivalent. To address these questions, we recruited data from close to the entire Korean population using HIRA data in which the Korean population is obliged to be registered. Our results indicated that future CHD risk defined by the need for coronary RVs in subjects with T2DM was not a recent CHD equivalent. Furthermore, using HRs, we found that the magnitude of need for RVs was significantly different depending on the presence of T2DM and/or recent CHD. To the best of our knowledge, this is the largest study of its kind performed to date and the first performed exclusively in an Asian population to evaluate future CHD risk according to the presence of T2DM and/or recent CHD. Our results support the recent notion that it is both scientifically and clinically inadequate to pursue a one-size-fits-all strategy for patients with T2DM with regard to cardiovascular risk estimation [17,27].

Results from previous studies on the magnitude of CHD risk associated with diabetes or prior CHD appear inconsistent. Most recently, Hadaegh et al. reported that new and known T2DM was a CHD risk equivalent by analyzing data from a population-based cohort study of 2,267 men and 2,931 women over a mean follow-up period of 7.6 years [5]. By contrast, a recent cohort study from 750 Caucasian patients showed that vascular risk was much lower in patients with T2DM without pre-existing significant coronary artery disease evaluated by coronary angiography [11]. These discrepancies might be attributed to the several factors including different study designs, diverse study populations, different follow-up duration, and diverse definition of baseline CHD among studies, as well as the marked advances in medications for managing multi-factorial risks of CHD such as renin-angiotensin system blockers, statins and anti-platelet agents. Our analysis differs from many others because we used health information from the whole population using a nationwide claims database, thereby avoiding selection bias and providing a large enough sample to examine CHD risk across a broad range of ages. Furthermore, we defined the need for RVs using validated codes for coronary RVs (i.e., PCI and/or CABG), so there is no doubt about the development of CHD.

In general, diabetes predisposes to incident CHD, and CHD is the leading cause of death among diabetic patients [28,29]. After MI, diabetics have a more rapid atherosclerotic process than non-diabetic patients with MI [30]. Similarly, the presence of T2DM conferred a higher risk of need for RVs in subjects with pre-existing CHD in our study (Table 2). However, diabetic patients without recent CHD seemed to have a lower risk of future CHD events defined by the need for RVs than non-diabetic patients with recent CHD, at least in our Korean population.

Diabetes duration is a potent risk factor for coronary events in patients with T2DM [31,32], which might explain why studies of those with more advanced diabetes yield higher population estimates of CHD, whereas those including newly diagnosed patients yield lower estimates [31,33]. In line with these previous findings, although we could not know the exact duration of T2DM in our population, established T2DM showed a higher event rate of need for RVs than new-onset T2DM (Fig 1B). Furthermore, the age- and sex-adjusted HR (95% CI) of established T2DM for the need for RVs was significantly higher than that of new-onset T2DM (1.36, 95% CI; 1.31–1.42) (Table 3).

Diabetes raises the risk of CHD to a greater extent in women than in men, although the reason for this difference is not known [24]. Numerous prospective cohort studies demonstrate that diabetes is a stronger risk factor for CHD in women than in men, with age-adjusted CHD mortality rates 3- to 7-times higher in diabetic women than in non-diabetic women [3,34], and 2- to 3-times higher in diabetic men than in non-diabetic men [35,36]. When we reanalyzed the rates of need for RVs and calculated age-adjusted HRs for the need for RVs after stratifying subjects by age and sex, the risk gaps between subjects with T2DM only and those with recent CHD only became smaller in women (Fig 2). Our results are in agreement with earlier studies showing that diabetes raises the risk of CHD to a greater extent in women than in men [9,37], although it was not as equivalent as recent CHD. Furthermore, the risk gap between the two groups in women (T2DM only vs. recent CHD only) was smallest in the 50–59 year age group (i.e., perimenopausal women), while the risk gap between the two groups showed a linear decreasing trend with age in men (Fig 2). However, the actual need for RVs in the T2DM only group increased with increasing age in both sexes (Fig 2). These findings suggest that we should manage diabetic subjects in a different way based on their sex, age and menopausal state (in case of women).

Our study has some limitations that should be taken into account. Firstly, we did not include incident CHD events that were not candidates for coronary RVs (PCI or CABG) or that were fatal. There remains a possibility that the incident CHD rate in diabetic subjects in this study was underestimated, as CHD is often asymptomatic in these patients until the onset of MI or sudden cardiac death [38]. Secondly, the actual previous CHD might have underestimated, as we ascertained the status of recent CHD during recent two years (i.e., 2007 to 2008). Moreover, based on the definition of T2DM in this study, we could not guarantee whether T2DM only group had no previous CHD before 2007. And it is also unclear that this study assessed a risk classification of T2DM against primary or secondary prevention for CHD. Thirdly, because the HIRA database could not provide the information according to the emergency of CHD and/or on the evaluated stage of their coronary ischemia, we could not discriminate between emergent coronary RVs and elective RVs through the database contrary to other previous study, in which clinical hard endpoints (i.e., MI and/or unstable angina) needed for emergent coronary RVs were adopted as incident CHD [17]. It might be a major bias in such clinical settings. Fourthly, we could not adjust for differences in cardiovascular risk factors at baseline among groups. Although we were unable to adjust for differences in cardiovascular risk factors at baseline such as lifestyle factors including smoking status and dietary habits, the presence of hypertension and/or dyslipidemia, and concurrent cardio protective medications that subjects were taking, multivariate adjustment may not be critical because such adjustments only reduced the HR for CHD events slightly in previous studies [4,13]. In our study, a substantial decrease would be required for the difference in risk of need for RVs to become insignificant among groups (Table 2, Table 3 and Table 4). Another important point is the narrow width of the confidence intervals in our study (Table 2, Table 3 and Table 4). Finally, the definition of T2DM based on the use of OADs could be problematic as this could exclude subjects with undiagnosed diabetes or on diet and exercise without OADs. Furthermore, we did not include subjects with T2DM on insulin only when defining the prevalence of T2DM, as claims for insulin are not based on the duration of insulin prescription. However, through this exclusion, type 1 diabetes mellitus (T1DM) might have been excluded as much as possible. Lastly, the relatively short follow-up period in this study (approximately 4 years) may interfere with our ability to draw definite conclusions.

The strength of the present study is that it is a large population-based cohort study including nearly all Koreans with T2DM and/or recent CHD. We believe a key advantage of the study is that it uses a primary endpoint derived using the HIRA database in Korea, since HIRA provides quality-controlled, reliable data.

In conclusion, this analysis of data from the nationwide claims database revealed that T2DM did not have a recent CHD equivalent risk in the Korean population. These results suggest that an appropriate strategy for the CHD risk stratification in diabetic patients should be adopted to manage this population.
